# Decentralizing acute stroke reperfusion therapies: moving from structural centralization to organizational equity

**DOI:** 10.3389/fneur.2026.1815354

**Published:** 2026-04-28

**Authors:** Carla Vera-Cáceres

**Affiliations:** 1 Neurology Department, General Hospital of Fuerteventura, Puerto del Rosario, Spain; 2 University of Las Palmas de Gran Canaria, Las Palmas de Gran Canaria, Spain

**Keywords:** decentralizing, endovascular treatment (EVT), health care organization, inequity access, stroke, stroke care access, stroke treatment disparities, urban–rural

Acute ischemic stroke remains one of the major causes of disability and mortality worldwide (1). The introduction of mechanical thrombectomy has reshaped the management of stroke, establishing endovascular treatment (EVT) as standard care for eligible patients. Despite strong clinical evidence for EVT and its time-dependent benefit (2), access to these therapies continues to vary substantially across regions. Many health systems still concentrate access to EVT in tertiary centers, often requiring interhospital transfers for patients initially presenting to secondary or regional hospitals.

This viewpoint contends that we should no longer consider the current centralization of acute stroke reperfusion therapies, particularly EVT, as a structurally mandatory organization. Instead, it represents a modifiable organizational choice. The purpose is not to argue against centralization itself but to support the selective expansion of qualified thrombectomy-capable centers in specific regions within coordinated hub-and-spoke stroke systems while maintaining referral pathways and quality standards.

## Stroke is too prevalent and treatable to be centralized

Unlike rare or ultra-specialized conditions, stroke is a common medical emergency. Incidence data indicate that every general hospital will inevitably face acute stroke cases on a daily or weekly basis. However, in many regions, patients eligible for reperfusion therapies must be transferred long distances to access comprehensive stroke centers (CSC).

Endovascular treatment has been firmly established as an effective therapy for large vessel occlusion (LVO), particularly in anterior circulation stroke, where randomized clinical trials have consistently shown benefit. More recently, long-term outcomes from the ATTENTION randomized clinical trial also confirmed a functional advantage in basilar artery occlusion (3). Ongoing refinement of devices and procedural techniques may further broaden EVT applicability to selected medium-vessel occlusions, although randomized evidence for routine EVT in MeVO remains evolving (4). As durable benefit is demonstrated across vascular territories, including some of the most devastating forms of ischemic stroke, the central challenge is no longer proving efficacy but ensuring that access to endovascular therapy is equitable without avoidable delay.

Each interhospital transfer adds time, reducing the potential benefit of reperfusion. In this context, geographic location or a simple postal code can become a determinant of neurological outcome. The principle that therapeutic effectiveness declines rapidly with time is particularly evident among patients living in geographically isolated or remote regions. Importantly, remoteness should not be mistaken for low population density or low stroke incidence; these regions often face structural barriers to stroke care despite a substantial and predictable stroke burden.

Centralization has historically supported quality and procedural refinement. Concentrating EVT in high-volume centers helped secure the technique's development and standardized outcome monitoring during its early adoption (5). However, as evidence has matured, continued reliance on highly centralized models warrants careful reconsideration. No time-dependent pathology should tolerate systematic delays of several hours attributable to organizational design.

## Infrastructure and system design

In many health systems, angiography suites are already present in secondary and regional hospitals, which can empower local teams and inspire confidence in decentralizing stroke services. Angiography suites are widely available and routinely support complex cardiovascular and other interventional procedures, suggesting that the technical environment necessary for EVT is often already present beyond CSC. For example, in Spain, more than 300 angiography suites operate nationwide, but only approximately 20% are currently involved in delivering EVT (6). Such disparities suggest that the primary limitation may not be the infrastructure itself but rather its integration into stroke care systems.

In many regions, EVT remains restricted to a small number of centers, often regardless of geographic location. Where transfer distances are substantial, this structural concentration reduces the proportion of eligible patients treated within optimal therapeutic windows. Instead of focusing only on technological capability, the main challenge lies in how stroke systems are organized. Improving access to reperfusion requires strategic regional planning and optimization of system performance.

## Equity and health policy considerations

In a highly time-dependent disease, delays are not merely operational inconveniences; they have measurable clinical consequences. Persistent geographic variation in access to reperfusion therapies, especially EVT, raises important questions about equity. These patterns reflect what may be described as a “geographical stroke penalty, “the systematic reduction in therapeutic benefit associated with distance from thrombectomy-capable centers. Geographic disparities in access to EVT, limited thrombectomy availability across many regions, and persistent delays in interhospital transfer are all well-recognized contributors to inequity in acute stroke care (7–9).

Despite that, comprehensive stroke care remains available in only a limited number of hospitals. This distribution invites reconsideration of whether existing organizational models adequately support equitable access to the population.

Strategic documents such as the Stroke Action Plan for Europe have explicitly highlighted the need to reconsider how stroke centers and stroke units are distributed across urban and rural regions and how reperfusion therapies can be delivered more rapidly, safely, and effectively at a systems level (10). Consistent with this perspective, real-world experience from Lithuania suggests that coordinated stroke system redesign can improve access, as implementation of a national stroke policy was associated with increased use of reperfusion therapies and better coordination across hospitals within a national stroke network (11).

In the same way, international guidance also increasingly emphasizes the importance of equitable access to stroke therapies. As populations age and stroke incidence rises globally, reliance on limited tertiary care capacity may become increasingly constrained. Establishing clear regional standards, particularly for rural and remote areas, for acceptable transfer times and reperfusion rates could support more transparent evaluation of system performance. When such goals are not met or are unsustainable, health systems should consider reassessing and adapting organizational models, including the potential role of structured decentralization. Selective decentralization guided by geography, population density, and quality monitoring may represent one pathway forward.

## Neurointerventional shortage as a modifiable system barrier

Workforce limitations are usually cited as a major barrier to expanding access to EVT. Regional differences in the number of qualified neurointerventionists often exacerbate existing inequalities. Workforce capacity, however, is not a fixed constraint; rather, it reflects policy decisions about professional pathways, education, and accreditation.

Training structures can be changed. Harmonized competency and increased endovascular training exposure during neurology residency may gradually increase capacity while preserving procedural safety and quality. Training pathways in EVT may also warrant reconsideration, including the development of accelerated training routes in stroke endovascular treatment. Current shortages of neurointerventionists should encourage strategic investment in education and training models aligned with current epidemiologic demand, rather than providing an excuse for perpetual centralization with an unsustainable EVT lack of access.

## Discussion

**Figure 1:**
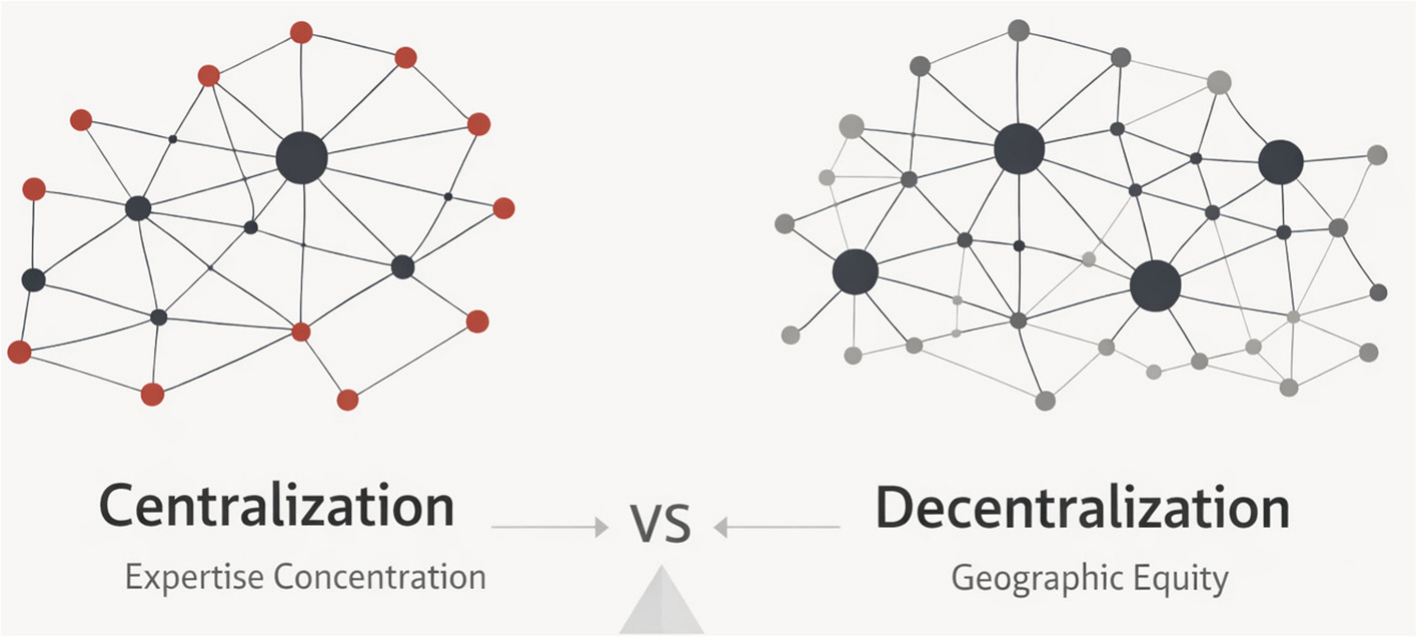
Conceptual comparison between centralized and decentralized models of acute stroke care. Centralization concentrates expertise within a limited number of hubs, whereas decentralization distributes thrombectomy-capable centers geographically, aiming to improve timely and equitable access to endovascular treatment.

Quality, safety, efficiency, and equity should be balanced in the organization of acute stroke reperfusion treatment. This balance can be conceptually understood as a continuum between centralized and decentralized organizational models (Figure 1). The safe application and refinement of mechanical thrombectomy have been facilitated by centralization. During the early stages of EVT expansion, concentrated expertise in comprehensive stroke centers enabled structured training, outcome monitoring, and procedural improvement. This historical contribution should be acknowledged explicitly, as higher-volume centers have been associated with faster workflows and better outcomes, and CSCs remain essential for training, governance, and quality assurance (10).

However, a reevaluation of policy is necessary due to the persistence of geographic disparities in access to acute stroke treatment and the growing evidence of EVT benefit. The question now is not whether EVT is effective, but whether stroke care systems are set up to benefit those most likely to require it.

Decentralization does not mean indiscriminate expansion of EVT or dilution of expertise. Instead, it may involve the selective formation of qualified EVT regional centers, guided by geographic realities, population density, and transparent quality metrics. Elements like structured accreditation, shared protocols, and continuous audits can help maintain safety and standards. Decentralization should represent not fragmentation but an adaptive and secure change.

In practice, this may involve regional thrombectomy-capable stroke centers working in close coordination with CSC hubs, using shared protocols; tele-stroke or remote expert support where appropriate; predefined referral pathways for complex cases; formal regional transfer agreements, credentialing standards; and continuous audit of procedural and clinical outcomes.

Importantly, different models may be needed across countries and regions. Urban areas with short transfer times may justify highly centralized systems, while geographically dispersed or isolated areas may require alternative options to reduce delays. A single central-system approach does not suit all population realities.

In conclusion, stroke is a time-dependent disease with effective reperfusion therapies available, and equitable access to these treatments requires resilient organizational models. Centralization has been crucial in the implementation of EVT in comprehensive stroke centers; whether it should remain the dominant configuration in all contexts remains a matter of debate. The goal is not to replace centralized systems but to refine them through network-based expansion where structural delays prevent equitable access. In stroke care, minutes matter.
